# Intestinal Inflammation, Dysfunction of Intestinal Digestion, and Disorder in the Intestinal Microbiota and Their Metabolites Caused by Oral *Microcystis* Exposure in Common Carp (*Cyprinus carpio*)

**DOI:** 10.3390/biology15010038

**Published:** 2025-12-25

**Authors:** Mengya Lou, Changqin Jing, Xin Liu, Yiyi Feng, Xiaoyu Li

**Affiliations:** 1 College of Life Sciences, Henan Normal University, Xinxiang 453007, China; lmyzpz@163.com (M.L.); lxxhh1281@163.com (X.L.); 2020040@htu.edu.cn (Y.F.); 2 School of Life Science and Technology, Henan Medical University, Xinxiang 453003, China; jingchangqin@126.com

**Keywords:** *Microcystis aeruginosa*, common carp, gut microbiota, intestinal digestion, intestinal inflammation

## Abstract

Cyanobacterial blooms are a common water pollution phenomenon worldwide now. Among them, *Microcystis* blooms occur most frequently internationally, and most of them can produce hepatotoxins—microcystins (MCs). When the bloom occurs in water bodies, common carp, as the top consumers in the aquatic food chain, inevitably ingest a large amount of cyanobacteria. The effects on the intestinal digestion, immunity, and microbiota of the carp remain to be revealed. In the present study, 21-day gavage exposure to *Microcystis* paste (equivalent to 378.25 μg/kg of MC-LR) was conducted on juvenile carp mainly to investigate the adverse effects of MCs on the fish intestinal digestion, immunity, and microbiota. The results showed that *Microcystis* exposure caused intestinal barrier damage, intestinal structure destruction, and increased permeability. Analysis of the activities of three main intestinal digestive enzymes showed that *Microcystis* exposure affected the intestinal digestion of fish. Targeted metabolome analysis and 16S rRNA sequencing showed that the abundance of opportunistic pathogens such as Proteobacteria and Aeromonas increased in the exposed fish, and the contents of intestinal metabolites lipopolysaccharide (LPS) and trimethylamine oxide (TMAO) increased. However, the abundance of Firmicutes and Bacillus that produce short-chain fatty acids (SCFAs) was significantly reduced. These results indicated that *Microcystis* exposure could imbalance the intestinal flora and increase the abundance of Gram-negative bacteria, thereby activating the inflammatory response in the common carp. In summary, oral *Microcystis* exposure by the common carp can negatively affect fish digestion, immunity, and flora homeostasis of the fish intestinal tract. This study also provides a theoretical basis for a comprehensive evaluation of the beneficial effects of SCFAs on regulating metabolic disorders, activating lipid catabolism, and nutrient utilization of fish in aquaculture.

## Simple Summary

Cyanobacterial blooms in aquatic environment are often accompanied by the release of microcystins (MCs), which can threaten the health of aquatic organisms. In the present study, intestinal barrier damage, oxidative stress, and inflammation were found to occur in the common carp after the ingestion of *Microcystis aeruginosa*. At the same time, the digestive function of the carp decreased, and the homeostasis of intestinal flora was disordered, accompanied by content reduction in the beneficial metabolites from fish intestinal flora. These findings provide an important theoretical basis for a comprehensive evaluation of the intestinal toxicity of MCs on fish.

## 1. Introduction

In fish, the gut is not only able to participate in the digestion and absorption of food, but also serves as a component of the immune system [1,2]. The gut of fish contains many lymphocytes, macrophages, eosinophils, and neutrophils. As the first barrier to contact with the outside, the intestinal mucosal immune system has been developed in fish to protect the body from potential threats such as toxic substances and dangerous microorganisms [3]. Intestinal flora can provide important nutrients for fish through the bacterial metabolism. For instance, SCFAs can promote the maturation of the intestinal mucosal immune system.

It is home to a variety of microbes in the gut, including bacteria, fungi, and viruses, which together make up a rich microbial community capable of maintaining intestinal integrity, metabolism, and immune regulation [4,5]. Intestinal probiotics can regulate intestinal mucosal inflammation, stimulate angiogenesis, are conducive to repairing damaged intestines, and promote intestinal health. In addition, probiotics could prevent tumor metastasis by regulating the expression of tight junction proteins, inhibiting the expression of metalloproteinases and tumor epithelial–mesenchymal transition. However, intestinal flora imbalance can cause intestinal barrier damage, leading to the occurrence of an inflammatory response [6]. For instance, after exposure to carbendazim, the abundance of Aeromonas significantly increased, thereby leading to intestinal tract inflammation in fish [7].

It is reported that microbiota-driven metabolites (including SCFAs, LPS and TMAO) are important signaling molecules for microbiota–host coupling [8,9]. LPS can cross the damaged intestinal barrier through pro-inflammatory factors to cause inflammation and promote the activation of immune cells through the Toll-like receptor 4 (TLR4) signaling pathway [10]. Studies have shown that oral LPS exposure not only changes the intestinal secretion and the composition of intestinal mucin and glycoprotein in carp, but also triggers inflammatory infiltration of intestinal tissue [11]. The imbalance of intestinal flora will destroy the intestinal barrier and increase the permeability of the intestinal wall, causing its metabolites, such as LPS, to directly enter the blood vessels through the intestinal wall. This process may increase the immune response of distant organs and intensify the release of nitric oxide and inflammatory factors. At the same time, dietary structure is also a key factor regulating the composition of intestinal flora and the production of metabolites [12]. A high-fat diet causes the intestinal microbiota to metabolize complexes containing trimethylamine groups to form trimethylamine (TMA), which is converted to TMAO in hepatocytes by the flavin-containing monooxygenase 3 (FMO3) [13]. It increases the expression of adhesion molecules, induces endothelial dysfunction and promotes apoptosis of vascular endothelial cells, thereby increasing the incidence of atherosclerosis and cardiovascular diseases [14]. Intestinal flora can produce metabolites by degrading and fermenting non-starch polysaccharides, thereby affecting the host’s immune system [15]. Certain fish microbiomes can degrade carbohydrates [16]. For example, treatment of carp with β-glucan in vivo causes changes in the composition of intestinal microbiota, leading to changes in SCFA production [17]. Gut microbes ferment indigestible oligosaccharides and dietary fiber to SCFAs [18,19], which can beneficially regulate the gut in a targeted manner [20,21]. SCFAs mainly include acetic acid, propionic acid, butyric acid, pentanoic acid, and other branched short-chain fatty acids. They can not only regulate the structure of intestinal flora and energy metabolism and maintain the integrity of intestinal epithelial structure [22], but also regulate the pH value in the intestine, improve intestinal function, and inhibit intestinal inflammation. They have potential roles in obesity [23], diabetes [24], and cancer [25]. SCFAs mediate G protein-coupled receptors (GPCRs) to coordinate signals involved in the physiological regulation of the host lipid metabolism and inflammation [8,26]. In particular, butyrate can inhibit the activation of nuclear factor kappa-B (NF-κB), enhance the peroxisome proliferator-activated receptor (PPAR) signaling pathway, and inhibit the expression of cytokines secreted by monocytes and lymphocytes. It has been shown that the exposure of zebrafish to cadmium reduces the relative abundance of SCFA-producing gut microbiota, resulting in a decrease in acetic acid concentration [27]. At present, SCFA-producing intestinal microorganisms have also been identified, such as *Bacteroides* [28], *Clostridium*, *Bifidobacterium* [29], and *Lactobacillus* [30]. Therefore, as the structure of the microbiota changes, the species and content of SCFAs will also change.

The eutrophication of water bodies has led to the explosion of cyanobacterial blooms in freshwater, accompanied by the release of cyanotoxins, especially microcystin (MC), mainly produced by *Microcystis* [31]. Fish living in water with low MC concentrations for a long time will be frequently damaged by toxic *Microcystis*. Related studies have shown that microcystin-LR (MC-LR) is transported to the zebrafish intestine via organic anion transporting polypeptides (Oatps), causing intestinal membrane enzyme dysregulation and oxidative stress [32]. The intestinal microbiota of freshwater fish (such as carp) is significantly influenced by water quality and feed. The intestinal microbiota, as a key component of the digestive system, is closely related to the health of the organism. The intake of algae may affect the composition and relative abundance of the microbial flora, thereby reducing the fish’s resistance to pathogenic bacteria and lowering their immune performance [33]. Therefore, by exploring the correlation between intestinal digestive enzymes and the species and abundance of intestinal flora, as well as the content of SCFAs in microbial metabolites, we can provide a better understanding for the intestinal mucosal immune balance and the prevention of inflammation.

## 2. Materials and Methods

### 2.1. Animals and Feeding

The experimental animals used in this study were juvenile carp (27.1 ± 1.6 g) purchased from a Yellow River Carp Farm in Henan Province, China. The fish were reared in an indoor running aquaculture system with a water temperature of 26.0 ± 1.0 °C and a light cycle of 14 h light and 10 h dark. The fish were fed regularly twice a day, the water was changed every two days, and the fish could be used for experiments after fourteen days of continuous acclimatization.

### 2.2. Microcystis Exposure and Sample Collection

In this experiment, the medium of *Microcystin aeruginosa* was removed by centrifugation at 7000 rpm to obtain algal slurry. After repeated freezing and thawing, the algal slurry was used for *Microcystis* exposure by gavage to common carp. The healthy domesticated carp were randomly divided into two groups, with 15 fish placed in each tank, and each group was set up with three replicates. All water quality indicators remained within the stable range of physiological adaptation of carp throughout the experimental period, without significant fluctuations or exceedances. The experimental group (MC group) was gavaged with 0.4 mL of *Microcystis* paste (equal to 378.25 μg/kg of MC-LR as determined by ELISA [34]) every 3 days. The control group (CK group) was given the equal volume of normal saline by gavage. This process was maintained for 21 days. When sampling on days 3, 7, 14, and 21, the carp were starved for 24 h, and tail vein blood was collected from 10 carps from each group using a 1 mL sterile syringe. After centrifugation, the supernatant was used for LPS and TMAO content determination. After blood collection, the fish were placed on ice for rapid dissection to collect the intestine, and 0.5 cm of the left and right midgut was placed in 4% paraformaldehyde for intestinal histological structure observation. The midgut was rinsed with normal saline, and the intestinal contents were collected for the detection of intestinal flora. The remaining intestinal samples were flash frozen in liquid nitrogen and stored at −80 °C for the determination of related gene expression, physiological and biochemical indicators, and intestinal metabolites. All experimental procedures were approved by the Ethics Committee of Henan Normal University (Approval number: HNSD-2024-11-25).

### 2.3. Pathological Examination

Three intestinal tissue samples were randomly selected from each group for the preparation of HE staining sections. Measurement of intestinal muscle layer thickness, villous height, and width was performed with an optical microscope (OLYMPUS, Tokyo, Japan) and ImageView 4.11 software.

### 2.4. Real-Time Quantitative RCR

Using an RNAiso plus kit (Takara, Dalian, Liaoning, China), extraction of total intestinal tissue RNA was conducted. RNA concentration (quality assessed as A260/A280 and A260/A230) was determined by a NanoDrop One (Thermo Scientific, Wilmington, DE, USA) spectrophotometer and RNA integrity was examined by electrophoresis on a 1% agarose gel [35]. Total RNA of the samples was reverse-transcribed into cDNA by HiFiScript cDNA Synthesis Kit (CWBIO, Beijing, China). Real-time PCR was performed according to the SYBR^®^ Green qPCR Mix Kit (MonAmp, Suzhou, China). Specific primers for the relevant genes are shown in Supplementary Table S1. After primer, specificity was verified by melting curves and the *EF-1α* gene was selected as an internal reference for normalized gene expression.

### 2.5. 16S rRNA Sequencing of Gut Microbiota

ABclonal DNA polymerase and specific primer 338F (5′-barcode+ACTCCTACGGGAGGCAGCA-3′) were used to extract genomic DNA from carp intestinal tissues. 806R (5′-GGACTACHVGGGTWTCTAAT-3′) was amplified by PCR [36]. The detailed methods are provided in Supplementary Methods S3.

### 2.6. Intestinal Metabolites Assay

Six intestinal tissue samples were randomly selected from each group for metabolite analysis. The contents of LPS and TMAO in carp serum were detected by ELISA. Based on liquid chromatography-mass spectrometry (LC-MS), seven major SCFAs in the intestine of carp were targeted for detection. The detailed methods are provided in Supplementary Methods S4 and S5.

### 2.7. Statistical Analysis

The experimental data were initially summarized with Excel 2021 software and statistically analyzed with SPSS 23.0 software. Normal distribution and homogeneity of variance were tested by Shapiro–Wilk test and Levene test. GraphPad Prism 9 software was used to visualize the data, one-way ANOVA was used to analyze the significance of the data, and the results are presented as mean ± standard error. Significant and highly significant differences between groups were expressed as * *p* < 0.05 and ** *p* < 0.01.

## 3. Results

### 3.1. Pathological Changes in the Intestines of Common Carp Following Microcystis Exposure

**Figure 1:**
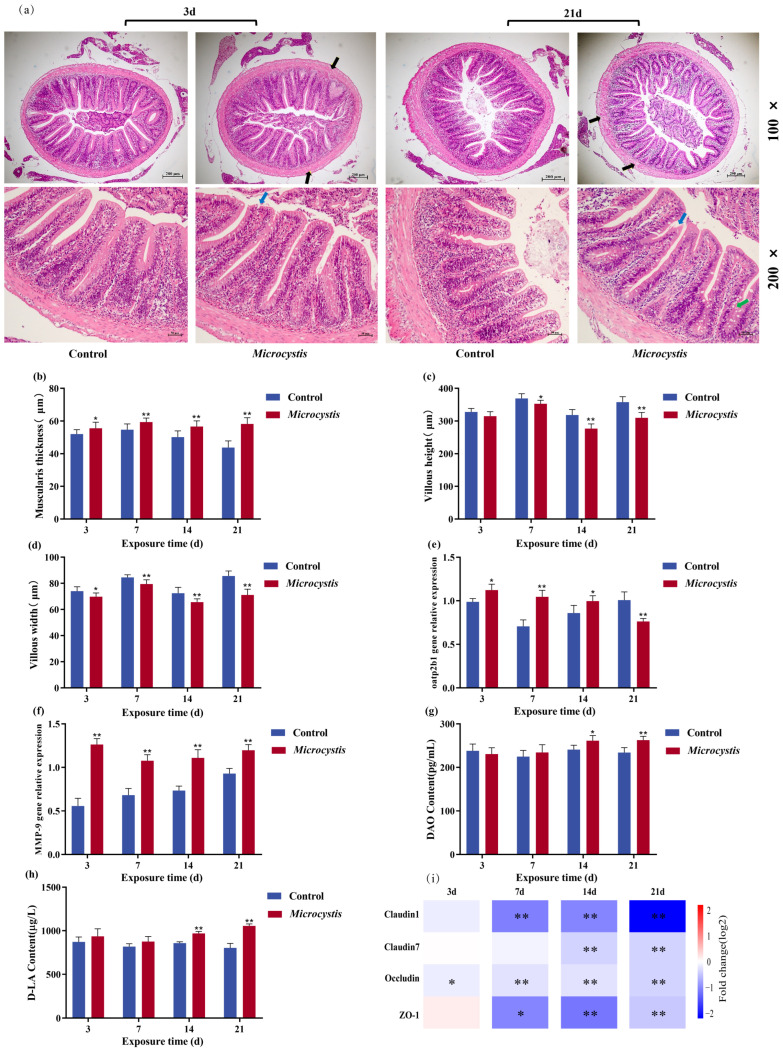
The effects of *Microcystis* exposure on the intestinal barrier. (**a**) Intestinal tissue sections at 7 and 21 days (Scale: 100 μm; 50 μm). (**b**) Muscularis thickness. (**c**) Villus height. (**d**) Villus width. Black arrow: loose bowel wall; Blue arrow: intestinal villus injury; Green arrow: increased number of goblet cells. (**e**) Gene expression of *Oatp2b1*. (**f**) Gene expression of *MMP-9*. (**g**) DAO content. (**h**) D-LA content. (**i**) Gene expression of tight junction proteins. Bars represent the mean ± SE (*n* = 3). Asterisks denote significant difference between the *Microcystis*-treated group and the control (* *p* < 0.05; ** *p* < 0.01).

The integrity of intestinal structure and function is crucial for the growth and development of the organism [37]. The results of HE staining showed that the intestinal structure of the carp in the control group was intact, the intestinal wall was smooth, and the intestinal villi were arranged neatly. However, loosening of the intestinal wall and varying degrees of villus injury were observed in the exposed fish intestines, and the number of goblet cells increased at 21 days (Figure 1a). Meanwhile, the thickness of the base layer increased markedly during the exposure period. The villus height showed a marked decrease from day 7, while the villus width decreased markedly throughout the exposure period (Figure 1b–d).

### 3.2. Gene Expression of Intestinal Oatp2b1, Matrix Metalloproteinase-9 (MMP-9), and Tight Junction Proteins

The gene expression of *Oatp2b1* was significantly increased at 3, 7, and 14 days, but it was significantly decreased at 21 days due to prolonged injury of intestine (Figure 1e). The expression of *MMP-9* increased markedly during the exposure period, and the increase was more obvious from day 3 to day 7 (Figure 1f). Except for slightly up-regulated expressions of *Occludin* and *Claudin7* on day 3, the expressions of *Claudin1*, *Occludin*, *Claudin7*, and *ZO-1* were significantly decreased during the exposure period (Figure 1i). This result suggests that the increased expression of *MMP-9* may aggravate the degradation of tight junction proteins and form a vicious cycle of “injury–inflammation”.

### 3.3. Intestinal Permeability

The changes in D-LA and DAO contents can reflect the integrity of intestinal mucosal mechanical barrier and the imbalance of intestinal flora. DAO and D-LA contents gradually increased during exposure and were significantly higher than controls from day 14 (Figure 1g,h), indicating that the intestinal permeability was increased and the composition of intestinal flora was disordered.

### 3.4. Effect of Microcystis Exposure on Intestinal Digestive Capacity

**Figure 2:**
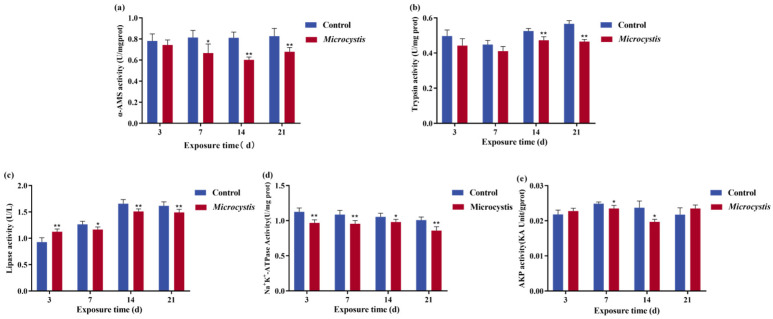
Effect of *Microcystis* exposure on intestinal digestive capacity. (**a**) α-AMS activity. (**b**) Trypsin activity. (**c**) Lipase activity. (**d**) Na^+^/K^+^-ATPase activity. (**e**) AKP activity. Bars represent the mean ± SE (*n* = 3). Asterisks denote significant difference between the *Microcystis*-treated group and the control (* *p* < 0.05; ** *p* < 0.01).

The activity of α-Amylase (α-AMS) in the fish intestine after *Microcystis* exposure was significantly decreased except on day 3. From the 14th day of exposure, the activity of trypsin decreased significantly. However, there was a transient rise in lipase activity in the early period; the activity decreased significantly with increasing exposure time (Figure 2a–c). For brush border enzyme activity, the activity of Na^+^/K^+^-ATPase in the intestine was significantly reduced with the increase in exposure days (Figure 2d). Intestinal AKP activity fluctuated during the exposure period and only showed a significant downward trend from day 7 to day 14 (Figure 2e).

### 3.5. Intestinal Oxidative Stress

**Figure 3:**
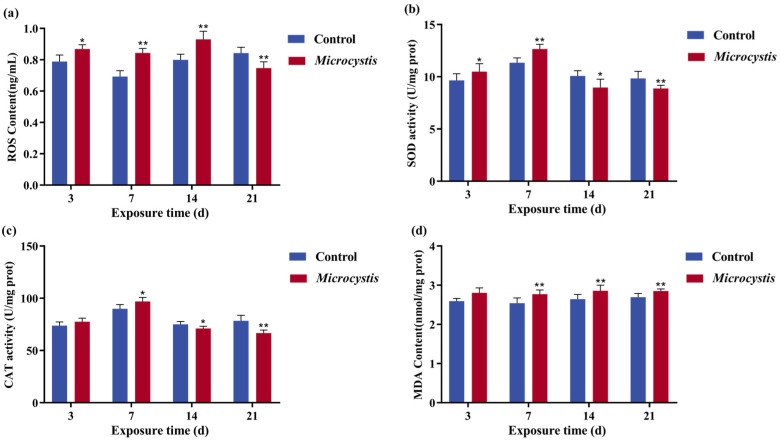
Effects of *Microcystis* exposure on intestinal oxidative stress level. (**a**) ROS content. (**b**) SOD activity. (**c**) CAT activity. (**d**) MDA content. Bars represent the mean ± SE (*n* = 3). Asterisks denote significant difference between the Microcystis-treated group and the control (* *p* < 0.05; ** *p* < 0.01).

After *Microcystis* exposure, ROS content was markedly increased at 3, 7, and 14 days, which resulted in a transient compensatory increase in SOD and CAT activities from days 3 to 7 (Figure 3a,b). When continuously exposed to oxidative damage, the content of ROS significantly decreased at 21 days, and the activities of CAT and SOD began to decrease significantly at 14 and 21 days (Figure 3a–c). MDA content was higher than that of the control during all exposure periods but did not reach a significant level at 3 days (Figure 3d).

### 3.6. Intestinal Inflammation

**Figure 4:**
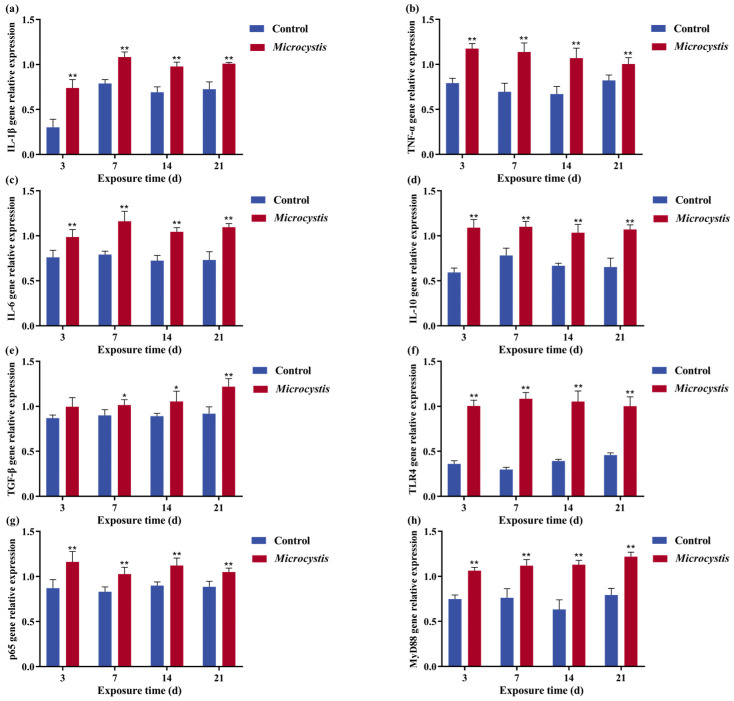
The effects of *Microcystis* exposure on the relative gene expression of *IL-1β* (**a**), *TNF-α* (**b**), *IL-6* (**c**), *IL-10* (**d**), *TGF-β* (**e**), *TLR4* (**f**), *p65* (**g**), and *MyD88* (**h**). Bars represent the mean ± SE (*n* = 3). Asterisks denote significant difference between the *Microcystis*-treated group and the control (* *p* < 0.05; ** *p* < 0.01).

The gene expression levels of pro-inflammatory cytokines interleukin-6 (*IL-6*), interleukin-1β (*IL-1β*), *TNF-α*, anti-inflammatory cytokines transforming growth factor-β (*TGF-β*), and interleukin-10 (*IL-10*) (except on the 3rd day) were significantly increased after *Microcystis* exposure (Figure 4a–e). In the immune signaling pathway, the gene expression level of *TLR4*, myeloid differentiation factor 88 (*MyD88*), and RelA (*p65*) were significantly increased (Figure 4f–h).

### 3.7. Effects of Microcystis Exposure on Intestinal Microbes in Common Carp

#### 3.7.1. Analysis on the Characteristics of Intestinal Flora

**Figure 5:**
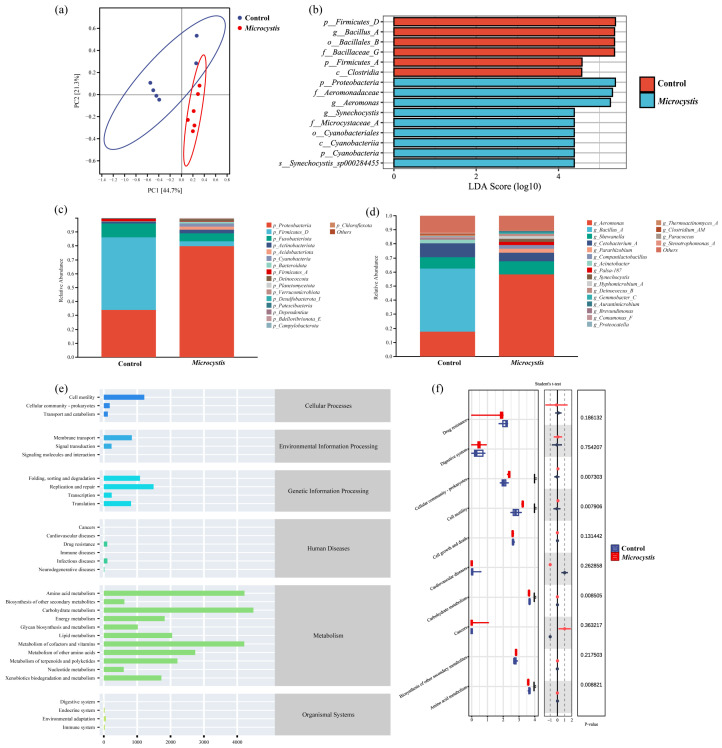
Effects of *Microcystis* exposure on intestinal microflora at 7 days. (**a**) Principal Coordinates Analysis (PCoA). (**b**) Linear discriminate analysis (LDA) score of LEfSe at all levels (LDA > 4.0). (**c**) Analysis of species composition at the phylum level. (**d**) Analysis of species composition at the genus level. (**e**) Relative abundance of KEGG secondary functional level. (**f**) KEGG secondary function level differences.

A total of 1683 ASVs were obtained from 12 samples by 16S rRNA high-throughput sequencing. The obtained microbial sequences were subjected to amplicon sequence variants (ASVs) clustering analysis under the condition of 100% sequence similarity. There were 22 phyla, 31 classes, 89 orders, 146 families, 276 genera, and 358 species in fish intestinal flora. The dilution curve of carp intestinal flora tended to be flat, indicating that sequencing had covered almost all bacteria, which could truly reflect the structure and diversity of bacterial communities (Figure S1a). The CV of sample variation was less than 20%, indicating that the sequencing depth was uniform and the comparability was good. The control group and the *Microcystis*-exposed group had 911 and 1009 ASVs, respectively, with a common number of 209 (Figure S1b). The Beta diversity analysis by Bray–Curtis distance showed that the two groups were significantly separated (Figure 5a and Figure S1c), suggesting that *Microcystis* exposure can markedly change the structure of intestinal microflora in carp.

The coverage, richness, diversity, and evenness of gut microbes were analyzed by Goods Coverage, Chao1, Shannon, and Simpson indices. The Goods Coverage index of each group was close to 100%, indicating that the species coverage in the community was high and the sequence analysis result was reliable. With the proliferation of opportunistic pathogens and the increase in the proportion of single dominant bacteria, Chao index decreased slightly. The Shannon index and Simpson index were increased, but the difference was not obvious. It indicates that *Microcystis* exposure may change the richness and diversity of gut microbiota.

#### 3.7.2. Species Composition of Intestinal Flora

Proteobacteria, Firmicutes, and Fusobacteriota were the dominant species at the phylum level in the fish intestine. The relative abundance of Proteobacteria (34.07%) increased significantly, while the relative abundance of Firmicutes (53.71%) decreased significantly (Figure 5c). At the genus level, *Aeromonas* (17.61%), *Bacillus*, and *Shewanella* were the dominant genera in the gut microbiota. Compared with the control group, *Aeromonas* (17.61%) was significantly increased, while *Bacillus* (44.96%) was significantly decreased (Figure 5d).

As shown in Figure 5b and Figure S1d, the differential distribution of intestinal flora in the two groups was analyzed by LDA Effect Size (LEfSe), and the species with marked differences in abundance between the groups were screened. Using LDA > 4.0 as the threshold, the flora with significant differences between the different groups were found. The control group was significantly enriched with Firmicutes, *Clostridia*, *Bacillus*, etc. Proteobacteria, Cyanobacteria (such as *Synechocystis*), *Aeromonas*, and *Shewanella* were significantly enriched in the *Microcystis* treatment group.

#### 3.7.3. KEGG Function Prediction

Based on the sequence abundance of ASVs in the samples, the abundance of each functional category represented by intestinal microorganisms was calculated and compared with the corresponding pathway information of ASVs in the KEGG database. The results indicate that the KEGG first-level pathways annotated by ASVs of the differential flora in the *Microcystis*-exposed group mainly included the metabolism, genetic information processing, cellular processes, and environmental information processing; most of the functions belonged to the metabolome (77.13%). The carbohydrate metabolism, the amino acid metabolism, the metabolism of cofactors and vitamins, and the lipid metabolism were the dominant functional pathways at the secondary level (Figure 5e). Among the top twenty pathways in abundance, four functional pathways were significantly changed in the *Microcystis* exposed group. Among them, the abundance of cellular community-prokaryotes and cell motility pathways increased significantly, while the abundance levels of carbohydrate metabolism and amino acid metabolism pathways decreased significantly (Figure 5f). Further analysis on the third-level pathways related to glucose and the lipid metabolism showed that after MC entered the intestine as an external toxin, bile acid biosynthesis and ubiquinone and other terpenoid-quinone biosynthesis were significantly increased. This indicates an enrichment of microbes associated with toxin degradation, thereby regulating microcystin degradation. However, the abundance of functional pathways related to fatty acid biosynthesis and glycolysis/gluconeogenesis were significantly decreased (Table 1).

### 3.8. Effects of Microcystis Exposure on Intestinal Metabolism of Common Carp

#### 3.8.1. LPS and TMAO from Intestinal Flora

**Figure 6:**
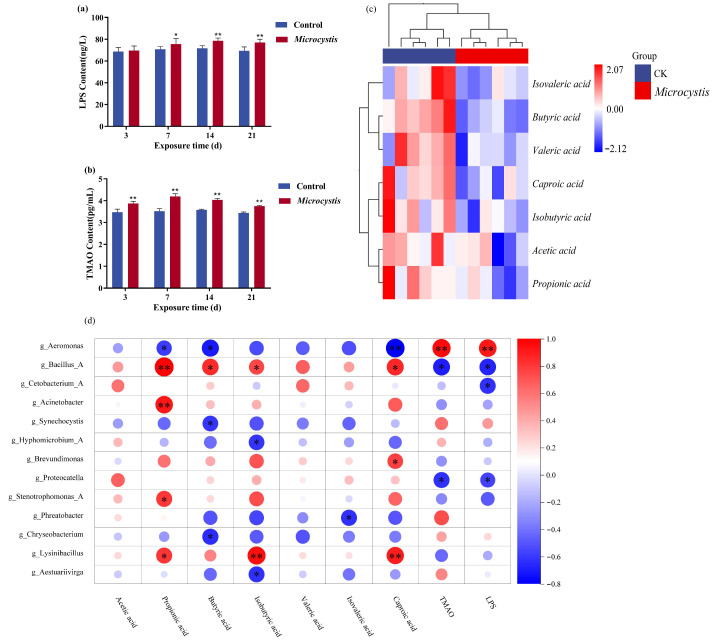
Intestinal metabolome and correlation analysis. (**a**) LPS content. (**b**) TMAO content. (**c**) Heatmap for hierarchical cluster analysis of SCFAs. (**d**) Spearman correlation analysis of SCFAs and gut microbiota. Bars represent the mean ± SE (*n* = 6). Asterisks denote significant difference between the *Microcystis*-treated group and the control (* *p* < 0.05; ** *p* < 0.01).

After exposure to *Microcystis*, the contents of LPS and TMAO, harmful metabolites of the gut microbiota, were significantly increased, but LPS content only slightly increased on day 3, which did not reach a significant level (Figure 6a,b). This result indicates an increase in intestinal permeability and a disorder of the microbiota.

#### 3.8.2. SCFAs Level

Based on the data analysis in Supplementary Table S2, Euclidean and hierarchical clustering longest distance methods (Complete Linkage) were used to calculate the distance and cluster analysis of the content values of seven SCFAs. The contents of SCFAs in the intestine of carp showed a downward trend after 7 days of *Microcystis* exposure. Among them, except for acetic acid, the contents of the other six SCFAs decreased significantly (Figure 6c).

#### 3.8.3. Correlation Analysis Between Gut Microbial Metabolites and Microbiota Abundance

The correlation between microbiota and metabolites at 7 days of *Microcystis* exposure was analyzed using Spearman’s correlation coefficient, and it was known that propionic acid, butyric acid, isobutyric acid, and caproic acid were negatively correlated with *Aeromonas*, *Synechocystis*, and *Hyphomicrobium* in Proteobacteria. They were positively correlated with beneficial bacteria such as *Bacillus* and *Lysinibacillus* in Firmicutes. And butyric acid was also negatively correlated with *Chryseobacterium* of Bacteroidetes.

For TMAO and LPS, *Aeromonas* had a significant positive correlation with them, while *Bacillus* and *Proteocatella* in Firmicutes had a significant negative correlation. Meanwhile, the amount of LPS was also negatively correlated with *Cetobacterium*. Several studies have shown that under adverse conditions or disease states, such as inflammatory bowel disease (IBD), *Cetobacterium* abundance is decreased and accompanied by an increased LPS level or exacerbation of inflammation (Figure 6d).

## 4. Discussion

The eutrophication of water bodies has led to the explosion of cyanobacterial blooms in freshwater and the release of microcystins, threatening the health of aquatic organisms. Fish gut is a natural immune barrier to direct exposure to foreign substances and pathogens [38]. The disruption of the structure and function of the gut results in the production of various cytokines and inflammatory mediators, along with bacterial and endotoxin translocation [39]. Therefore, the aim of this study is to investigate the effects of microcystin on intestinal injury from the aspects of histopathology, serum biochemistry, and intestinal flora metabolism.

Analysis of intestinal structural integrity is an important indicator to assess the intestinal toxicity of pollutants. It has been shown that *Microcystis aeruginosa* exposure leads to lysis and exfoliation of intestinal epithelial cells and an increase in the number of goblet cells in zebrafish gut [40]. Goblet cells can protect epithelial cells and defend against foreign pathogens by secreting mucus [41]. Consistent with this experiment, our result showed that after *Microcystis* exposure, the intestine of common carp was obviously damaged, for example, the height and width of villi were reduced, and the muscular layer was thickened. In addition, in order to resist the invasion of toxins, the demand for mucus secretion and the number of goblet cells increased. This result suggests that *Microcystis* exposure leads to the destruction of intestinal structure and impairment of barrier function in common carp.

Similarly, intestinal tight junction proteins are also considered as markers to evaluate the integrity of the tissue barrier [42]. It has been shown that climbazole can destroy the intestinal tight junctions of grass carp, thereby causing intestinal damage [43]. MMP-9, a closely related matrix metalloproteinase, is involved in the degradation and remodeling of extracellular matrix (ECM) homeostasis. In this experiment, the gene expression of *Claudin1*, *Claudin7*, *Occludin*, and *ZO-1* were remarkably decreased, but *MMP-9* expression was significantly increased. This indicates that during exposure, extracellular matrix degrades, intestinal permeability increases, and mucosal integrity is damaged. Related studies have shown that Oatp2b1 can participate in the transmembrane transport of MC-LR and is essential for toxin transport [32]. MC can be actively ingested into intestinal epithelial cells through Oatp2b1 and accumulated in the cells. However, with the increase in *MMP-9* expression, the tight junction protein was degraded and the expression of *Oatp2b1* was inhibited, which aggravated the accumulation of MC and induced apoptosis of intestinal epithelial cells [29]. As an intracellular enzyme in intestinal mucosal cells, the release of DAO is positively correlated with the degree of intestinal injury. D-LA is a metabolite of intestinal flora [44], which can penetrate tissues from the intestinal lumen when the barrier is broken. In this study, the contents of intestinal DAO and D-LA were significantly increased from 14 days of exposure, which further suggested that *Microcystis* exposure increased intestinal permeability, impaired intestinal barrier, and disordered intestinal flora.

Food digestion and absorption can provide energy for fish and maintain the stability of physiological functions [45]. Carp can produce glycerol monoesters and free fatty acids from triglycerides by emulsification of bile acids and hydrolysis of fatty acids, which are absorbed by intestinal epithelial cells [46]. In this study, the toxin stimulated bile acid secretion and transiently promoted lipase release at the early time of *Microcystis* exposure, but the activities of α-AMS, trypsin, and lipase decreased remarkably with the increase in exposure time. The digestive function of the intestine is also related to the brush border structure. Brush border exists on the free surface of intestinal epithelial cells, which is composed of many neatly arranged microvilli, which contribute to nutrient absorption and pathogen defense. Due to the injury of intestinal epithelial cells and villus, the protective effect of brush border will fail, and the digestive function will decline [47]. In addition, there are abundant AKP and ATPase in the brush border cell membrane, which are involved in the reabsorption function of the cells. In this experiment, Na^+^/K^+^-ATPase and AKP activities were markedly reduced during exposure. Thus, it can be concluded that *Microcystis* exposure can lead to a decrease in the activities of digestive enzymes and brush edge enzymes in carp, thereby reducing fish digestion and absorption capacity.

The destruction of intestinal barrier caused by pollutant exposure is accompanied by the occurrence of intestinal inflammation in fish [6]. When MC enters the fish body, it is recognized by the TLR4 pattern recognition receptor, initiating the innate immune response, and then activating MyD88, NF-κB, and mitogen-activated protein kinase (MAPK) signaling cascade, thereby inducing the production of TNF-α, IL-1β, IL-6. It was shown that MC-LR exposure can significantly increase the expression levels of *TLR4* and *MyD88* genes in the spleen of zebrafish [48]. With the release of pro-inflammatory cytokines and the activation of the immune system, anti-inflammatory pathways (such as IL-10/TGF-β) are simultaneously activated to prevent excessive injury. In the present study, the anti-inflammatory factors peaked relatively late compared with the pro-inflammatory factors, and there was a lag. During Microcystis exposure, the gene expressions of *IL-1β*, *TNF-α*, *IL-6*, *IL-10*, *TGF-β*, *TLR4*, *MyD88*, and *p65* in the intestinal tract of the common carp were significantly increased. *TGF-β* was not significantly changed at 3 days, but its expression was significantly up-regulated with increasing exposure time, thereby promoting tissue repair. It indicates that *Microcystis* exposure can trigger the occurrence of intestinal inflammation in carp.

Inflammatory response is often associated with oxidative stress [49]. When there is an inflammatory reaction, it is often accompanied by immune cell infiltration and release of a large amount of ROS to attack the tissue, leading to the aggravation of damage and amplification of inflammatory effect [50]. Studies have shown that oxidative stress can cause damage, immunosuppression, and death of fish cells and tissues, and lead to damage of Na^+^/K^+^-ATPase structure. In the present study, ROS content was significantly elevated during exposure except at 21 days, which presumably caused apoptosis. SOD and CAT, as key antioxidant enzymes, can remove ROS and its breakdown products and protect cells from oxidative damage. The generation of ROS activates SOD expression, and intestinal SOD and CAT activities increased significantly before 14 days of exposure, but continued oxidative damage induced the destruction of the heme prosthetic group of CAT, causing a decrease in activity. This result indicates that excessive ROS can cause disorders in the intestinal redox system. In addition, excessive ROS can attack cellular membrane, proteins, and DNA, leading to lipid peroxidation and tissue damage [51]. MDA content is positively correlated with intestinal villus injury, which can reflect the degree of oxidative damage. In this study, MDA content increased significantly during the period of *Microcystis* exposure, except on day 3, indicating that *Microcystis* exposure triggered oxidative stress and lipid peroxidation in the carp gut.

Long-term oxidative stress and inflammation are often closely related to intestinal flora imbalance [52]. It has been reported that exposure to pollutants can lead to the destruction of the balance of intestinal microorganisms. Therefore, studying the interaction between pollutants and intestinal microorganisms has become a new direction in toxicology research [53]. As the main players in metabolism, gut microbes are crucial in maintaining the body’s health. The imbalance of intestinal microorganisms can damage the normal physiological function, leading to inflammatory response and metabolic abnormality. In this study, Proteobacteria, Firmicutes, and Fusobacteriota were the dominant populations in fish gut. PCoA and NMDS analyses revealed a significant separation between the two groups, indicating that *Microcystis* exposure could cause a significant difference in the intestinal microbial community structure of carp. Proteobacteria dominate the gut microbiota of fish, and like Fusobacteria, changes in their abundance are often related to intestinal inflammation [54,55], while Firmicutes contribute to intestinal fatty acid absorption [56]. In this study, on the 7th day of exposure, the toxin effect was obvious, and the inflammation and oxidative stress reached the peak. At this time, the flora was in the period of upheaval, with the explosive proliferation of opportunistic pathogen such as Proteobacteria, while the beneficial bacteria such as Firmicutes decreased significantly. In this study, *Microcystis* exposure led to significant differences in the dominant flora at the genus level. *Aeromonas*, a Gram-negative bacterium, is a known intestinal pathogen in teleost fishes. It can colonize the intestinal mucosal surface, disrupt host nutrient absorption, and cause intestinal diseases. *Bacillus* is a kind of Gram-positive bacterium and it can produce resistant endospores, which are conducive to maintaining intestinal ecological balance. It was observed that the abundance of *Aeromonas* and *Shewanella* remarkably increased, while that of *Bacillus* remarkably decreased in this study, indicating that *Microcystis* exposure could disrupt the homeostasis of gut microbiota, increase the Gram-negative bacteria, and activate intestinal inflammatory response.

Gut microbial metabolites can profoundly affect host health through energy supply, immune regulation, and nerve signal transmission [57,58]. Metabolic imbalance may lead to gastrointestinal diseases, metabolic syndrome, and neurodegenerative diseases [59,60]. LPS can stimulate the body to produce immune cascades and cause toxic pathological activities [61]. In addition, TMAO is also a gut-derived microbiota-associated metabolite, which affects cholesterol transport and reduces the production of very-low-density lipoprotein (VLDL). TMAO increases the accumulation of cholesterol in blood vessels and promotes the formation of atherosclerotic plaques when released into the blood. In this study, serum LPS and TMAO levels were significantly increased in the MC-treated fish, and correlation analysis showed that their levels were positively correlated with the abundance of *Aeromonas*, indicating that *Microcystis* exposure can trigger an increase in the abundance of Gram-negative bacteria, which led to LPS and TMAO release and activation of inflammatory cascade. In the intestinal tract, specific flora can also produce SCFAs by digestion of dietary fiber [62], which can provide a lot of energy for colon cells, reduce local inflammation, and promote the production of antimicrobial peptides by cells. SCFAs help to maintain the intestinal barrier and reduce intestinal permeability, thus playing a positive role in intestinal mucosal immunity. Therefore, the reduction in SCFAs-producing bacteria could result in inflammation in the fish gut [63]. MC-LR can reduce the abundance and diversity of SCFAs-producing bacteria in the gut, thereby reducing the content of SCFAs [29]. In this study, except for acetic acid, the contents of six other SCFAs in the intestine were observably reduced after *Microcystis* exposure. This is related to the increase in the abundance of opportunistic pathogens such as *Aeromonas*, which are associated with inflammation and intestinal barrier damage, and a decrease in the abundance of beneficial commensal bacteria such as *Bacillus* and *Chrysobacter* (Figure 6d). Consistent with the KEGG function prediction on the intestinal microorganisms, the decrease in SCFAs due to *Microcystis* exposure was related to the inhibition of the lipid metabolism and the significant reduction in fatty acid biosynthesis in the tertiary metabolic pathways.

## 5. Conclusions

*Microcystis* exposure can cause intestinal tissue damage, disrupt the normal expression of tight junction proteins, and trigger intestinal inflammation and oxidative stress.

Moreover, *Microcystis* exposure caused the inhibition of the activities of intestinal brush edge enzymes and digestive enzymes and the disruption of the integrity of the intestinal barrier, leading to weakened digestive function and intestinal flora imbalance. After *Microcystis* exposure, the abundance of opportunistic pathogens such as Proteobacteria increased, while the abundance of SCFAs-producing Firmicutes decreased, which leads to the reduction in SCFAs content and aggravates the occurrence of intestinal inflammation. This study provides a more comprehensive understanding of the health risks of cyanobacterial bloom and MC to fish as well as aquatic ecosystems.

The present study indicates that *Microcystis* and its toxins not only have hepatotoxicity on the common carp in water bodies with cyanobacterial blooms, but also have intestinal toxicity. Moreover, *Microcystis* and its toxins can cause changes in the intestinal flora and displacement of harmful products of the intestinal flora, and lead to an imbalance in intestinal immune homeostasis. These results suggest that *Microcystis* and its toxins may be important factors causing slow growth, weakened immunity, and enteritis in common carp living in water bodies with cyanobacterial blooms.

## Figures and Tables

**Table 1:** The KEGG tertiary functional pathways with significant differences.

KEGG Pathway	*p* Value	Decrease/Increase
Synthesis and degradation of ketone bodies	0.0489	↓
Fatty acid biosynthesis	0.0009	↓
Pentose and glucuronate interconversions	0.0195	↓
Primary bile acid biosynthesis	0.0113	↑
Glycolysis/Gluconeogenesis	0.0039	↓
Ubiquinone and other terpenoid-quinone biosynthesis	0.0016	↑
Citrate cycle (TCA cycle)	0.0136	↑
Secondary bile acid biosynthesis	0.0411	↑

Note. ↑, increased functional pathway abundance; ↓, decreased functional pathway abundance.

## Data Availability

Data supporting the reported results are contained within the article.

## Supplementary Materials

The following supporting information can be downloaded at: https://www.mdpi.com/article/10.3390/biology15010038/s1, Figure S1: Effects of Microcystis exposure on gut microbial communities at 7 days; Table S1: Primer sequences for synthetic genes; Table S2: The levels of seven short-chain fatty acids in the intestines of common carp.

## References

[1] Lokesh J., Fernandes J.M.O., Korsnes K., Bergh Ø., Brinchmann M.F., Kiron V. (2012). Transcriptional Regulation of Cytokines in the Intestine of Atlantic Cod Fed Yeast Derived Mannan Oligosaccharide or β-Glucan and Challenged with *Vibrio anguillarum*. Fish Shellfish Immunol..

[2] Fan Y., Pedersen O. (2021). Gut Microbiota in Human Metabolic Health and Disease. Nat. Rev. Microbiol..

[3] Niklasson L., Sundh H., Fridell F., Taranger G.L., Sundell K. (2011). Disturbance of the Intestinal Mucosal Immune System of Farmed Atlantic Salmon (*Salmo salar*), in Response to Long-Term Hypoxic Conditions. Fish Shellfish Immunol..

[4] Agus A., Clément K., Sokol H. (2021). Gut Microbiota-Derived Metabolites as Central Regulators in Metabolic Disorders. Gut.

[5] Weng Y., Huang Y., Qian M., Jin Y. (2024). Epoxiconazole Disturbed Metabolic Balance and Gut Microbiota Homeostasis in Juvenile Zebrafish. Pestic. Biochem. Physiol..

[6] Dong B., Moon H.-B. (2025). Toxicological Effects of Chemical Pesticides in Fish: Focusing on Intestinal Injury and Gut Microbial Dysbiosis. Pestic. Biochem. Physiol..

[7] Bao Z., Zhao Y., Wu A., Lou Z., Lu H., Yu Q., Fu Z., Jin Y. (2020). Sub-Chronic Carbendazim Exposure Induces Hepatic Glycolipid Metabolism Disorder Accompanied by Gut Microbiota Dysbiosis in Adult Zebrafish (*Daino rerio*). Sci. Total Environ..

[8] Kim S., Seo S.-U., Kweon M.-N. (2024). Gut Microbiota-Derived Metabolites Tune Host Homeostasis Fate. Semin. Immunopathol..

[9] Yang L., Wu Y., Yang J., Li Y., Zhao X., Liang T., Li L., Jiang T., Zhang T., Zhang J. (2024). *Lactiplantibacillus Plantarum P470* Isolated from Fermented Chinese Chives Has the Potential to Improve In Vitro the Intestinal Microbiota and Biological Activity in Feces of Coronary Heart Disease (CHD) Patients. Nutrients.

[10] Zhao Z., Ning J., Bao X., Shang M., Ma J., Li G., Zhang D. (2021). Fecal Microbiota Transplantation Protects Rotenone-Induced Parkinson’s Disease Mice via Suppressing Inflammation Mediated by the Lipopolysaccharide-TLR4 Signaling Pathway through the Microbiota-Gut-Brain Axis. Microbiome.

[11] Neuhaus H., Van der Marel M., Caspari N., Meyer W., Enss M., Steinhagen D. (2007). Biochemical and Histochemical Effects of Perorally Applied Endotoxin on Intestinal Mucin Glycoproteins of the Common Carp *Cyprinus carpio*. Dis. Aquat. Organ..

[12] Querio G., Antoniotti S., Geddo F., Levi R., Gallo M.P. (2023). Modulation of Endothelial Function by TMAO, a Gut Microbiota-Derived Metabolite. Int. J. Mol. Sci..

[13] Laryushina Y., Samoilova-Bedych N., Turgunova L., Kozhakhmetov S., Alina A., Suieubayev M., Mukhanbetzhanov N. (2024). Alterations of the Gut Microbiome and TMAO Levels in Patients with Ulcerative Colitis. J. Clin. Med..

[14] Geng J., Yang C., Wang B., Zhang X., Hu T., Gu Y., Li J. (2018). Trimethylamine N-Oxide Promotes Atherosclerosis via CD36-Dependent MAPK/JNK Pathway. Biomed. Pharmacother..

[15] López Nadal A., Ikeda-Ohtsubo W., Sipkema D., Peggs D., McGurk C., Forlenza M., Wiegertjes G.F., Brugman S. (2020). Feed, Microbiota, and Gut Immunity: Using the Zebrafish Model to Understand Fish Health. Front. Immunol..

[16] Kihara M., Sakata T. (2002). Production of Short-Chain Fatty Acids and Gas from Various Oligosaccharides by Gut Microbes of Carp (*Cyprinus carpio* L.) in Micro-Scale Batch Culture. Comp. Biochem. Physiol. A Mol. Integr. Physiol..

[17] Petit J., de Bruijn I., Goldman M.R.G., van den Brink E., Pellikaan W.F., Forlenza M., Wiegertjes G.F. (2022). β-Glucan-Induced Immuno-Modulation: A Role for the Intestinal Microbiota and Short-Chain Fatty Acids in Common Carp. Front. Immunol..

[18] Zhang D., Jian Y.-P., Zhang Y.-N., Li Y., Gu L.-T., Sun H.-H., Liu M.-D., Zhou H.-L., Wang Y.-S., Xu Z.-X. (2023). Short-Chain Fatty Acids in Diseases. Cell Commun. Signal..

[19] Li X., He M., Yi X., Lu X., Zhu M., Xue M., Tang Y., Zhu Y. (2024). Short-Chain Fatty Acids in Nonalcoholic Fatty Liver Disease: New Prospects for Short-Chain Fatty Acids as Therapeutic Targets. Heliyon.

[20] Liu C., Zhao L.-P., Shen Y.-Q. (2021). A Systematic Review of Advances in Intestinal Microflora of Fish. Fish Physiol. Biochem..

[21] Assan D., Kuebutornye F.K.A., Hlordzi V., Chen H., Mraz J., Mustapha U.F., Abarike E.D. (2022). Effects of Probiotics on Digestive Enzymes of Fish (Finfish and Shellfish); Status and Prospects: A Mini Review. Comp. Biochem. Physiol. B Biochem. Mol. Biol..

[22] Sivaprakasam S., Prasad P.D., Singh N. (2016). Benefits of Short-Chain Fatty Acids and Their Receptors in Inflammation and Carcinogenesis. Pharmacol. Ther..

[23] Tagliamonte S., Laiola M., Ferracane R., Vitale M., Gallo M.A., Meslier V., Pons N., Ercolini D., Vitaglione P. (2021). Mediterranean Diet Consumption Affects the Endocannabinoid System in Overweight and Obese Subjects: Possible Links with Gut Microbiome, Insulin Resistance and Inflammation. Eur. J. Nutr..

[24] Tao Z., Wang Y. (2025). The Health Benefits of Dietary Short-Chain Fatty Acids in Metabolic Diseases. Crit. Rev. Food Sci. Nutr..

[25] Zagato E., Pozzi C., Bertocchi A., Schioppa T., Saccheri F., Guglietta S., Fosso B., Melocchi L., Nizzoli G., Troisi J. (2020). Endogenous Murine Microbiota Member *Faecalibaculum rodentium* and Its Human Homologue Protect from Intestinal Tumour Growth. Nat. Microbiol..

[26] Pi Y., Fang M., Li Y., Cai L., Han R., Sun W., Jiang X., Chen L., Du J., Zhu Z. (2024). Interactions between Gut Microbiota and Natural Bioactive Polysaccharides in Metabolic Diseases: Review. Nutrients.

[27] Yang J., Li J., Zhang X., Zhou Q., Wang J., Chen Q., Meng X., Xia Y. (2023). Effects of Ecologically Relevant Concentrations of Cadmium on the Microbiota, Short-Chain Fatty Acids, and FFAR2 Expression in Zebrafish. Metabolites.

[28] He X., Qi Z., Hou H., Qian L., Gao J., Zhang X.-X. (2020). Structural and Functional Alterations of Gut Microbiome in Mice Induced by Chronic Cadmium Exposure. Chemosphere.

[29] Zhang Y., Jiang D., Jin Y., Jia H., Yang Y., Kim I.H., Dai Z., Zhang J., Ren F., Wu Z. (2021). Glycine Attenuates *Citrobacter rodentium* -Induced Colitis by Regulating ATF6-Mediated Endoplasmic Reticulum Stress in Mice. Mol. Nutr. Food Res..

[30] Li A., Ni W., Zhang Q., Li Y., Zhang X., Wu H., Du P., Hou J., Zhang Y. (2020). Effect of Cinnamon Essential Oil on Gut Microbiota in the Mouse Model of Dextran Sodium Sulfate-induced Colitis. Microbiol. Immunol..

[31] Gallet A., Halary S., Duval C., Huet H., Duperron S., Marie B. (2023). Disruption of Fish Gut Microbiota Composition and Holobiont’s Metabolome during a Simulated *Microcystis aeruginosa* (Cyanobacteria) Bloom. Microbiome.

[32] Li J., Chen C., Zhang T., Liu W., Wang L., Chen Y., Wu L., Hegazy A.M., El-Sayed A.F., Zhang X. (2019). ΜEvaluation of Microcystin-LR Absorption Using an in Vivo Intestine Model and Its Effect on Zebrafish Intestine. Aquat. Toxicol..

[33] Feng Y., Li L., Ma Q., Liu S., Wang P., Li X., Ma J. (2025). Effect of Microcystin-LR on Intestinal Microbiota, Metabolism, and Health of Zebrafish (*Danio rerio*). Sci. Total Environ..

[34] Zhao H., Sun K., Nan X., Ding W., Ma J., Li X. (2024). Hepatocyte Apoptosis Is Triggered by Hepatic Inflammation in Common Carp Acutely Exposed to Microcystin-LR or Chronically Exposed to Microcystis. Ecotoxicol. Environ. Saf..

[35] Shi L., Feng L., Jiang W.-D., Liu Y., Jiang J., Wu P., Kuang S.-Y., Tang L., Tang W.-N., Zhang Y.-A. (2016). Immunity Decreases, Antioxidant System Damages and Tight Junction Changes in the Intestine of Grass Carp (*Ctenopharyngodon idella*) during Folic Acid Deficiency: Regulation of NF-ΚB, Nrf2 and MLCK MRNA Levels. Fish Shellfish Immunol..

[36] Zhou L., Limbu S.M., Shen M., Zhai W., Qiao F., He A., Du Z.-Y., Zhang M. (2018). Environmental Concentrations of Antibiotics Impair Zebrafish Gut Health. Environ. Pollut..

[37] Vizcaíno A.J., López G., Sáez M.I., Jiménez J.A., Barros A., Hidalgo L., Camacho-Rodríguez J., Martínez T.F., Cerón-García M.C., Alarcón F.J. (2014). Effects of the Microalga *Scenedesmus almeriensis* as Fishmeal Alternative in Diets for Gilthead Sea Bream, Sparus Aurata, Juveniles. Aquaculture.

[38] Martens E.C., Neumann M., Desai M.S. (2018). Interactions of Commensal and Pathogenic Microorganisms with the Intestinal Mucosal Barrier. Nat. Rev. Microbiol..

[39] Lei X., Zhang D., Wang Q., Wang G., Li Y., Zhang Y., Yu M., Yao Q., Chen Y., Guo Z. (2022). Dietary Supplementation of Two Indigenous *Bacillus* spp. on the Intestinal Morphology, Intestinal Immune Barrier and Intestinal Microbial Diversity of *Rhynchocypris lagowskii*. Fish Physiol. Biochem..

[40] Qian H., Zhang M., Liu G., Lu T., Sun L., Pan X. (2019). Effects of Different Concentrations of Microcystis Aeruginosa on the Intestinal Microbiota and Immunity of Zebrafish (*Danio rerio*). Chemosphere.

[41] Dolan B., Ermund A., Martinez-Abad B., Johansson M.E.V., Hansson G.C. (2022). Clearance of Small Intestinal Crypts Involves Goblet Cell Mucus Secretion by Intracellular Granule Rupture and Enterocyte Ion Transport. Sci. Signal..

[42] Robinson B.D., Tharakan B., Lomas A., Wiggins-Dohlvik K., Alluri H., Shaji C.A., Jupiter D., Isbell C.L. (2020). Exploring Blood-Brain Barrier Hyperpermeability and Potential Biomarkers in Traumatic Brain Injury. Bayl. Univ. Med. Cent. Proc..

[43] Lu Z.-J., Shi W.-J., Ma D.-D., Zhang J.-G., Long X.-B., Li S.-Y., Gao F.-Z., Zhang Q.-Q., Ying G.-G. (2024). The Azole Biocide Climbazole Induces Oxidative Stress, Inflammation, and Apoptosis in Fish Gut. Sci. Total Environ..

[44] Shen S., Zhao J., Dai Y., Chen F., Zhang Z., Yu J., Wang K. (2020). Methamphetamine-Induced Alterations in Intestinal Mucosal Barrier Function Occur via the MicroRNA-181c/ TNF-α/Tight Junction Axis. Toxicol. Lett..

[45] Debnath S., Saikia S.K. (2021). Absorption of Protein in Teleosts: A Review. Fish Physiol. Biochem..

[46] Omer E., Chiodi C. (2024). Fat Digestion and Absorption: Normal Physiology and Pathophysiology of Malabsorption, Including Diagnostic Testing. Nutr. Clin. Pract..

[47] Maroux S., Coudrier E., Feracci H., Gorvel J.-P., Louvard D. (1988). Molecular Organization of the Intestinal Brush Border. Biochimie.

[48] Lin W., Guo H., Wang L., Zhang D., Wu X., Li L., Qiu Y., Yang L., Li D., Tang R. (2020). Waterborne Microcystin-LR Exposure Induced Chronic Inflammatory Response via MyD88-Dependent Toll-like Receptor Signaling Pathway in Male Zebrafish. Sci. Total Environ..

[49] Bellanti F., Coda A.R.D., Trecca M.I., Lo Buglio A., Serviddio G., Vendemiale G. (2025). Redox Imbalance in Inflammation: The Interplay of Oxidative and Reductive Stress. Antioxidants.

[50] Sahoo D.K., Heilmann R.M., Paital B., Patel A., Yadav V.K., Wong D., Jergens A.E. (2023). Oxidative Stress, Hormones, and Effects of Natural Antioxidants on Intestinal Inflammation in Inflammatory Bowel Disease. Front. Endocrinol..

[51] Muro P., Zhang L., Li S., Zhao Z., Jin T., Mao F., Mao Z. (2023). The Emerging Role of Oxidative Stress in Inflammatory Bowel Disease. Front. Endocrinol..

[52] Goyette P., Labbé C., Trinh T.T., Xavier R.J., Rioux J.D. (2007). Molecular Pathogenesis of Inflammatory Bowel Disease: Genotypes, Phenotypes and Personalized Medicine. Ann. Med..

[53] Qiu W., Liu T., Liu X., Chen H., Luo S., Chen Q., Magnuson J.T., Zheng C., Xu E.G., Schlenk D. (2022). Enrofloxacin Induces Intestinal Microbiota-Mediated Immunosuppression in Zebrafish. Environ. Sci. Technol..

[54] Garrett W.S., Gordon J.I., Glimcher L.H. (2010). Homeostasis and Inflammation in the Intestine. Cell.

[55] Liu C., Huang D., Sheng X., Zhu J., Dong S., Chen S., Wang Y., Tang A., Duan R., Yang Z. (2024). Integrated Physiological, Intestinal Microbiota, and Metabolomic Responses of Adult Zebrafish (*Danio rerio*) to Subacute Exposure to Antimony at Environmentally Relevant Concentrations. Ecotoxicol. Environ. Saf..

[56] Machate D.J., Figueiredo P.S., Marcelino G., Guimarães R.d.C.A., Hiane P.A., Bogo D., Pinheiro V.A.Z., Oliveira L.C.S.d., Pott A. (2020). Fatty Acid Diets: Regulation of Gut Microbiota Composition and Obesity and Its Related Metabolic Dysbiosis. Int. J. Mol. Sci..

[57] Wang Z., Dan W., Zhang N., Fang J., Yang Y. (2023). Colorectal Cancer and Gut Microbiota Studies in China. Gut Microbes.

[58] Zhu J., Liu X., Liu N., Zhao R., Wang S. (2024). Lactobacillus Plantarum Alleviates High-Fat Diet-Induced Obesity by Altering the Structure of Mice Intestinal Microbial Communities and Serum Metabolic Profiles. Front. Microbiol..

[59] Westfall S., Lomis N., Kahouli I., Dia S.Y., Singh S.P., Prakash S. (2017). Microbiome, Probiotics and Neurodegenerative Diseases: Deciphering the Gut Brain Axis. Cell. Mol. Life Sci..

[60] Sorboni S.G., Moghaddam H.S., Jafarzadeh-Esfehani R., Soleimanpour S. (2022). A Comprehensive Review on the Role of the Gut Microbiome in Human Neurological Disorders. Clin. Microbiol. Rev..

[61] Skočková V., Vašíček O., Sychrová E., Sovadinová I., Babica P., Šindlerová L. (2023). Cyanobacterial Harmful Bloom Lipopolysaccharides Induce Pro-Inflammatory Effects in Immune and Intestinal Epithelial Cells In Vitro. Toxins.

[62] Parada Venegas D., De la Fuente M.K., Landskron G., González M.J., Quera R., Dijkstra G., Harmsen H.J.M., Faber K.N., Hermoso M.A. (2019). Short Chain Fatty Acids (SCFAs)-Mediated Gut Epithelial and Immune Regulation and Its Relevance for Inflammatory Bowel Diseases. Front. Immunol..

[63] Zhao Y., Li S., Lessing D.J., Chu W. (2024). The Attenuating Effects of Synbiotic Containing *Cetobacterium somerae* and Astragalus Polysaccharide against Trichlorfon-Induced Hepatotoxicity in Crucian Carp (*Carassius carassius*). J. Hazard. Mater..

