# Supplementary material for: Intestinal Inflammation, Dysfunction of Intestinal Digestion, and Disorder in the Intestinal Microbiota and Their Metabolites Caused by Oral Microcystis Exposure in Common Carp (Cyprinus carpio)
Source: Biology (Basel). 2025 Dec 25;15(1):38. doi: 10.3390/biology15010038 (PMC12785017; doi:10.3390/biology15010038)
Supplement: Supplementary file 1 [file biology-15-00038-s001.zip › biology-4022656-supplementary.pdf]

Supplementary materials

Intestinal inflammation, dysfunction of intestinal digestion, and disorder in the intestinal microbiota and their metabolites caused by oral *Microcystis* exposure in common carp (*Cyprinus carpio*)

Mengya Lou[1], Changqin Jing [2], Xin Liu[1], Yiyi Feng[1], Xiaoyu Li[1, *]

1. College of Life Sciences, Henan Normal University, Xinxiang 453007, China.
2. School of Life Science and Technology, Henan Medical University, Xinxiang 453003, China.

[*] Corresponding author at: College of Life Sciences, Henan Normal University, Xinxiang 453007, China.
E-mail addresses: lixiaoyu65@263.net (X. Li).

Supplementary Methods S1

Batch culture of Microcystis aeruginosa

The *Microcystis* used in this study were collected from Nanwan Reservoir in China, August 2023 by using the No. 25 plankton net, and isolated and cultured by capillary separation method. The selected uncontaminated single *Microcystis* community was transferred to a sterile conical flask with BG11 medium and incubated at 25°C in a constant temperature incubator, with 1 500 lux of light intensity at a light and dark ratio of 16:8 h. The algal cells at the exponential growth phase were poured into a 10 L bucket, and an appropriate amount of medium was added to light-green color. The mouth of the bucket was blocked with a sterile cotton ball and put into an oxygen pump with a sterile filter membrane for oxygen.

Supplementary Methods S2

Determination of biochemical index and intestinal enzyme activity

After weighing the intestinal specimens, they were gently rinsed with 0.9% saline, blotted dry, and mechanically homogenized on ice by adding precooled saline at a ratio of weight (g) : volume (mL) = 1:9. In the low temperature centrifuge with 4 000 rpm centrifuge for 10 min, take that measured supernatant. The contents of D-lactic acid (D-LA), reactive oxygen species (ROS), and diamine oxidase (DAO) were detected by ELISA method. Intestinal α-Amylase (α-AMS), trypsin, lipase, alkaline phosphatase (AKP), sodium potassium ATPase (Na^+/K^+ ATPase), catalase (CAT), superoxide dismutase (SOD) activities, and malondialdehyde (MDA) content were measured using related commercial assay kits (Nanjing Jiancheng Institute, China).

Supplementary Methods S3

Sequencing and analysis of intestinal flora

Intestinal tissue genomic DNA was extracted using the Mag-Bind Soil DNA kit from OMEGA. The molecular size of extracted DNA was determined by 0.8% agarose gel electrophoresis, and DNA was quantified by Nanodrop. The 480 bp V3V4 region of the bacterial 16S rRNA gene was selected for sequencing. The V3V4 region of bacterial 16S rRNA gene with a length of about 480 bp was used for sequencing. ABclonal DNA polymerase and specific primers 338F (5 '-barcode+ACTCCTACGGGAGGCAGCA-3'), 806R (5 '-GGACTACHVGGGTWTCTAAT-3') were used for PCR amplification. TruSeq Nano DNA LT Library Prep kit (Illumina, Inc.) was used for library construction. For qualified libraries, 2×250 bp double-end sequencing was performed using NovaSeq 6 000 SP Reagent kit (500 cycles).

The original sequence data were decoded and primer excised using the demo and cutadapt plugin, and then the sequence was processed with data such as quality filtering, denoizing, splicing and Mosaic removal using the DADA2 plugin. The obtained sequences were merged according to 100% sequence similarity, and characteristic sequence ASVs and abundance data tables were generated. The Greengenes database was used for comparison and annotation. QIIME2 software was used for α and β diversity analysis. The metabolic function of the microbiota was predicted by PICRUSt2 on MetaCyc and KEGG databases. Based on the predicted results, PCoA analysis was performed on the functional unit at the functional level. At the same time, the differential metabolic pathways between the groups were screened according to adjPvalues and logFC, and the species composition of metabolic pathways was analyzed.

Supplementary Methods S4

Determination of LPS and TMAO contents by ELISA

(1) Dilution and adding sample to the Standard: Tail venous blood of carp was collected, left at 4 °C for 5 h, centrifuged at 4 000 rpm for 10 min, and the supernatant was taken and stored at -80 °C for use.

(2) Adding sample: Set blank wells separately (No sample and HRP-Conjugate reagent was added to the blank comparison wells, other each step operation is same). Add Sample dilution 40 μL to testing sample well, then add testing sample 10 μL (sample final dilution is 5-fold), add sample to wells, don't touch the well wall as far as possible, and gently mix.

(3) Incubation: After closing plate with closure plate membrane, the samples were incubated for 30 min at 37 °C.

(4) Configuration: 30 times or 20 times of washing solution diluted 30 times (or 20 times) with distilled water to reserve.

(5) Washing: Uncover Closure plate membrane, discard Liquid, dry by swing. Add washing buffer to every well, still for 30 s then drain, repeat 5 times, dry by pat.

(6) Adding enzyme: Add HRP-Conjugate reagent 50 μL to each well, except blank well.

(7) Incubation: Operation with (4).

(8) Washing: Operation with (6).

(9) Color development: Add Chromogen Solution A 50 μL and Chromogen Solution B to each well, evade the light preservation for 15 min at 37 °C.

(10) Stopping the reaction: Add Stop Solution 50 μL to each well, stop the reaction (the blue color change to yellow color).

(11) Assay: Take blank well as zero, read absorbance at 450 nm after adding Stop Solution and within 15 min.

(12) Calculation: Excel was used to calculate the regression equation according to the OD value of standard Wells and the concentration of standard materials. The OD value of the sample was substituted into the regression equation, and then multiplied

by the dilution factor to obtain the final sample concentration.

Supplementary Methods S5

Determination of intestinal SCFAs content

A total of 20 mg intestinal tissue samples (n=6) were taken after 7 days of exposure, then 800 μL extractant (containing internal standard) was added and vortexed for 60 s, crushed, and centrifuged at 4 000 rpm for 10 min at 10 °C. 40 μL of the supernatant was added and mixed with 20 μL of 200 mM (3NPH). Then, 20 μL 120 mM (EDC) • HCl-6% pyridine solution was added, and the reaction was shaken at 1 200 rpm for 30 min at 40 °C in a constant temperature metal instrument. After the reaction, the mixture was cooled at -20 °C for 30 min and centrifuged at 12 000 rpm and 4 °C for 10min. The supernatant was used for LC-MS after passing through a 0.22 μm filter membrane. The quantitative software OS (version: 4.7, SCIEX) was used to integrate the chromatographic peaks of all target substances, and the standard curve was constructed by the standard substance for quantitative analysis.

Table S1. Primer sequences for synthetic genes

Gene	Sequences of primers (5'-3')
β-actin-F	CCGTCAGGCAGCTGATAGCT
β-actin-R	GCTATGTGGCTCTTGACTTCG
Oatp2b1-F	GAAAAATGGTGACGCCAGATG
Oatp2b1-R	GGAGGGTGGCAGCTATCAAG
Claudin1-F	CCACACTTCCCTCCAGCAAT
Claudin1-R	TGTTGTCTCCGGCGTATGAG
Claudin7-F	CTTCTATAACCCCTTCACACCAG
Claudin7-R	ACATGCCTCCACCCATTATG
Occludin-F	GGAGAGACTGCCAACGATTT
Occludin-R	TTCGGCACACAGTTCCTTATAG
ZO-1-F	GTGCCTCCGTATGATGAGCA
ZO-1-R	TAGCCCGTGTCATTTCGCAT
MMP-9-F	CATAGGCACATGAAACGGGATG
MMP-9-R	TCAACACAGAGAATGGATGCTT
IL-1β-F	GTCGCATTGGCAACTCATGG
IL-1β-R	TGTACACCCGCTGGATTTGT
TNF-α-F	ACAACAATCAGGAAGGTGGAA
TNF-α-R	TGGAAAGACACCTGGCTGTA
IL-6-F	GCGTGTTTGATGTCCTTCACCA
IL-6-R	TTACAGCGTCCTGACCTGGTAC
IL-10-F	GACACCATTCTGCCAACAGC
IL-10-R	GCTGGCGATCTCAAAGGGAT
TGF-β-F	GGTCTGCGTCACATCAAACG
TGF-β-R	CCAGATGGTTCAGAGCAGAGAC
p65-F	AGAAGAGCAACGACACCACAA
p65-R	TTGTACGGCTGGTTCTTGGTT
TLR4-F	GGAAGTCCATCGCCTCCAACA

Gene	Sequences of primers (5’-3’)
TLR4-R	AGCGACACCAGGCACTATCAAT
MyD88-F	TGGAGGACAGTCGCCGAAATG
MyD88-R	TTGCCACTGTTGCCTCTGGAC

Table S2. The levels of seven short-chain fatty acids in the intestines of common carp

Groups	Indices						
	Acetic acid	Propionic acid	Butyric acid	Isobutyric acid	Valeric acid	Isovaleric acid	Caproic acid
CK	194.58±3.28	5.83±0.45	2.12±0.09	0.92±0.08	0.69±0.05	4.22±1.54	2.57±0.18
MC	188.79±5.54	5.13±0.40*	1.86±0.07**	0.80±0.06*	0.61±0.04*	2.36±0.77*	2.25±0.16*

Note: *$p < 0.05$.**$p<0.01$.

Figure S1. Effects of *Microcystis* exposure on gut microbial communities at 7 days.

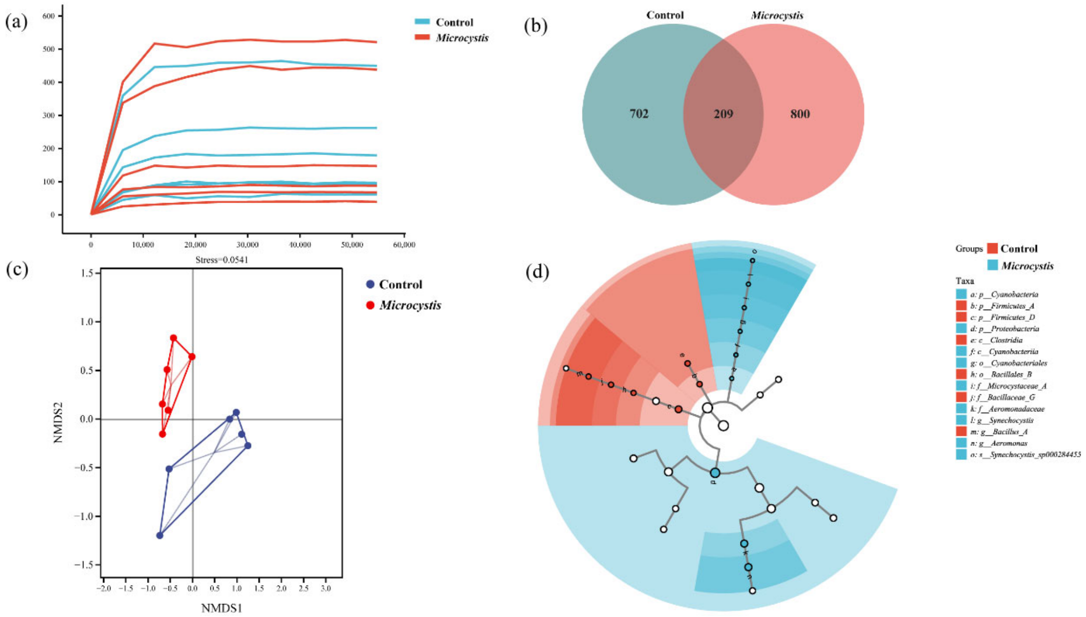

(a) Rarefaction curves. (b) Venn diagram. (c) Non-metric Multi-dimensional Scaling (NMDS). (d) Taxonomic cladogram obtained from LEfSe analysis of 16S rRNA sequences, each circle's diameter was proportional to the bacterial taxon abundance.
